# Regulation of inflammaging

**DOI:** 10.6026/973206300210571

**Published:** 2025-04-30

**Authors:** Francesco Chiappelli, Lily Fotovat, Allen Khakshooy, Oluwadayo Oluwadara, Jaden Penhaskashi

**Affiliations:** 1 Dental Group of Sherman Oaks, Sherman Oaks, CA 91403; 2 UCLA Center for the Health Sciences, Los Angeles, CA 90095; 3 Boston University, Boston, MA 02118; 4 Department of Internal Medicine, Valley Hospital Medical Center, Las Vegas, NV 89106; 5 Oluwadara Dental Corporation, 9612 East Foothill Blvd, Suite 100, Rancho Cucamonga, CA 91730; 6 Division of West Valley Dental Implant Center, Encino, CA 91316

**Keywords:** Acetylcholine (ACh), non-neuronal cholinergic system (NNCS), α7 nicotinic acetylcholine receptor (α7-nAChR), cholinergic anti-inflammatory pathway (CHAIP), resistance to inhibitors of cholinesterase-3 (RIC-3), MicroRNAs (miRs), inflammasome, pathogen-associated molecular patterns (PAMPs), host cell-associated damage-associated molecular patterns (DAMPs), pattern recognition receptors (PRRs), nucleotide leucine-rich repeat-containing receptors (NLRs), pyrin "cell death fold" (PYD) domain, caspase activation and recruitment domain (CARD), nucleotide-binding oligomerization domain (NOD), leucine-rich repeat (LRR), cyclic GMP-AMP synthase linked to a stimulator of interferon genes (cGAS-STING), metalloproteinase-8 (MMP8)

## Abstract

The non-neuronal cholinergic system plays an important role in the modulation of immunity and in particular the control of the inflammatory response. The cholinergic anti-inflammatory pathway is mediated principally by the α7-nicotinic acetylcholine receptor (α7-nAChR) and its receptor-specific chaperone, resistance to inhibitors of cholinesterase (RIC)-3 chaperone protein. The point is made in this writing that therapeutic stimulation of the non-neuronal cholinergic system via, for instance, transcutaneous vagal stimulation, pharmaco-therapeutic activation of α7-nAChR/RIC-3, or stimulation of the pathway by other means, including microRNA treatment interventions could down-regulate states of inflammation such as the cytokines storm and chronic systemic inflammation in aging - *viz*, inflammaging. It is possible and even likely that controlling inflammaging by intervening on the non-neuronal cholinergic system (NNCS) will be beneficial to a wide range of aging-related diseases, from type 2 diabetes and obesity to periodontal disease.

## Background:

The parasympathetic rest/digest division of the autonomic nervous system predominantly uses acetylcholine (Ach, *i.e.*, cholinergic response) to counterbalance the effects of the flight/fight sympathetic component of the autonomic nervous system. Anatomically, it consists of the craniosacral outflow, preganglionic cell bodies from one of two locations: brainstem (cranial nerves III, VII, IX, X) or sacral spinal cord (S2, S3 and S4), which synapse with postganglionic neurons generally located in or near the innervated organs. Centrally, neuronal cholinergic responses regulate attention, learning, sleep, arousal and conscious awareness among others. Peripherally, the non-neuronal cholinergic system (NNCS) controls important peripheral physiological responses as well from digestive (*e.g.*, peristalsis, relaxation of gastrointestinal sphincters) and cardiovascular (*e.g.*, heart rate, contractility and conduction, blood pressure) to immune processes. Muscarinic and nicotinic receptors modulate cholinergic function [1]. NNCS dysregulation - *i.e.*, dysregulated ACh synthesis and release, receptor mutations or polymorphisms, auto-antibodies against NNCS components - impact the etiology and progression of certain physiopathologies, including immune deregulation [2]. External stimuli and environmental factors have the potential to trigger NNCS. Cultural practices, exercise regimens, certain dietary intake and physical activities also can stimulate the NNCS and counteract stress and combat free radicals, thereby blunting inflammaging. It follows that NNCS is emerging as a promising therapeutic target in the quest to prevent or mitigate inflammation in general and inflammaging in particular and could come to play a pivotal role in the realm of personalized interventions (*i.e.*, patient-centered care), as noted below. To be clear, immune NNCS - *viz*, cholinergic regulation of myeloid and lymphoid immune cells and secondary lymphoid organs (*e.g.*, spleen) -, mediated principally by the α7-nicotinic acetylcholine receptor (α7-nAChR), controls and regulates inflammation as the cholinergic anti-inflammatory pathway (CHAIP) [3]. To be clear, α7-nAChR assembles into a homomeric receptor at the outer membrane and requires, for its functional maturation, the conserved endoplasmic reticulum-resident protein and nicotinic acetylcholine receptor-specific chaperone, resistance to inhibitors of cholinesterase (RIC)-3. α7-nAChR/RIC-3 is key to the immune cholinergic system in general and specifically to CHAIP [4]. One putative pathway by which α7-nAChR/RIC-3 regulates CHAIP is by modulating myeloid cell polarization, the process by which macrophages alter their function in response to micro environmental demands [5]. In brief, activated monocytes mature into M0 macrophages and then into either classically activated (M1) or alternatively activated (M2) macrophages, the latter being classified into M2a, M2b, M2c and M2d subclasses based on the expression of certain plasma membrane proteins, secretion of distinct cytokines and specific biological activities. Typically, lipopolysaccharides and TH1 cytokines (*e.g.*, IFN-γ) induce M1 polarization, whereas IL-4, IL-13, IL-10 and TGF-β induce M2 polarization [6, 7].

MicroRNAs (miRs) (*e.g.*, miR-375, miR-let7, miR-34a, miR-155, miR-124, miR-34a, miR-511-3p, miR-99a, miR-132 and miR-145-3p) also regulate macrophage polarization [7, 8]. It follows that identification of miR's related to macrophage polarization is critical to developing novel miR-targeted therapeutic strategies for immune regulation and control of inflammation [9]. Moreover and in the context of this discussion on inflammation regulation, the subclass M2b is particularly relevant due to its strong immune-regulatory and anti-inflammatory effects [10]. In point of fact, experimental in vitro findings indicate that α7-nAChR/RIC-3 stimulation appears to favor the generation of M2 macrophages - specifically M2b and M2d (*i.e.*, tumor-associated macrophage) - and apoptosis of their M1 counterparts [11]. Furthermore, manipulation of CHAIP - *viz*, by means of vagus nerve (Cranial X) stimulation [12], or promising pharmacotherapies targeting acting at the α7nAChR/RC-3 - may lead to clinically relevant attenuation of systemic inflammation. It is likely and even probable that systemic chronic inflammation in the elderly population (*i.e.*, inflammaging) results in part from aging-related attenuation in overall cholinergic tone and associated development and progression of the most common age-related diseases (ARDs), from type 2 diabetes to obesity, cardiovascular disease and kidney disease [13]. Indeed, chronic inflammation is implicated in a variety of diseases also independent of age, such as autoimmune diseases, malignancy [14] and viral infections. The activation of the hypothalamus-pituitary-adrenal (HPA) axis by these disease processes results in blunting of the immune response and ultimately deregulation of the immune system - *viz*, altered balance of TH1and proinflammatory cytokines vs. TH2 and anti-inflammatory mediators. The above has been well delineated in the Immune Restoration Inflammatory Syndrome (IRIS) in HIV patients [15], but emerging evidence proves similar with SARS-CoV2 infections and particularly the sequelae of the long-term post-acute CoViD-19 syndrome (PACS, Long Covid) [16, 17-18] . With respect to the latter, it is particularly important to note that the SARS-CoV2 spike protein shows remarkable affinity for α7-nAChR and, via its helical motif, downregulates expression of the receptor before it achieves RIC-3-mediated functional maturation [19]. If interference of α7-nAChR/RIC-3 by the SARS-CoV2 spike protein contributes to the cytokines storm, then NNCS stimulators in general, α7-nAChR/RIC-3 activators and related inflammatory cholinergic reflex therapeutic strategies could reduce, through interaction with diverse signaling pathways (*e.g.*, JAK2/STAT3/NF-κB), the risk of inflammatory disorders in CoViD-19 and in PACS [20, 21].

It is also the case, moreover, that inflammation is triggered by the inflammasome, the set of innate immune sensors that detect pathogen-associated molecular patterns (PAMPs), host cell-associated damage-associated molecular patterns (DAMPs), or free foreign peptidic or nuclear acid complexes, by forming specific cytosolic pattern recognition receptors (PRRs) in the host cells' cytosol and initiate an activation process that promotes the activation of caspase-1, which proteolytically cleaves and activates IL-1β and other pro-inflammatory cytokines and molecules. PRRs that drive the inflammasome principally consist of at least four species of nucleotide leucine-rich repeat-containing receptors (NLRs) containing a pyrin "cell death fold" (PYD) domain (*i.e.*, NLRPs) and/or a caspase activation and recruitment domain (CARD). In addition, NLRs also often express the nucleotide-binding oligomerization domain (NOD), essential to self-oligomerization and the C-terminal leucine-rich repeat (LRR), a critical ligand-recognition domain for other receptors or microbial ligands. NLRP3 is centrally relevant to immune recognition and surveillance by myeloid (*e.g.*, M1/M2 macrophages) and lymphoid cells (*e.g.*, TH1, TH17) throughout the lifespan [21]. Case in point, the inflammasome is involved in acute inflammation triggered by microbes (*e.g.*, parasites, bacteria, viruses), toxins, allergens and other pathogenic stimuli, as well as in sustained inflammation when innate immune sensing to such triggering stimuli is sustained, which can devolve in the cytokine storm. The inflammasome is also involved in chronic low-level systemic metabolic inflammation not triggered by pathogenic stimuli that manifests in the middle-aged and the elderly (*i.e.*, inflammaging) and contributes to aging-related diseases [22].

We [23] and others [24] have evoked the putative role of the cyclic GMP-AMP synthase linked to a stimulator of interferon genes (cGAS-STING) pathway in triggering transcriptional factors involved in the secretion of pro-inflammatory mediators particularly in the elderly hence enhancing inflammaging. We also discussed the role of certain miRs in inflammaging [25], as well as other inflammatory biomarkers, including the matrix metalloproteinase-8 (MMP8, *i.e.* neutrophil collagenase) [26]. Taken together, these lines of evidence lead us to suggest that therapeutic stimulation of NNCS (*e.g.*, via transcutaneous vagal stimulation), pharmacotherapeutic activation of α7-nAChR/RIC-3 and/or targeted miRs therapy, perhaps largely dictated by AI protocols such as immune tweening, machine learning and modeling inflammatory cascades as we outlined elsewhere [26, 27], show the promise, either individually or in concert, to diminish and control chronic inflammation in aging and senescence-related diseases by helping tailor treatment interventions targeted to inflammatory profiles. It is possible and even probable that an approach of this type will be particularly relevant for conditions such as periodontal pathologies since NNCS was reported in the periodontium and deemed accessible as therapeutic target in the treatment of periodontal disease [28], one among the most important aging-related chronic inflammatory pathologies [29].

## References

[1] Tiwari P (2013). Asian Pac J Trop Dis..

[2] Beckmann J, Lips KS (2013). Pharmacology..

[3] Duris K (2017). Curr Drug Deliv..

[4] Treinin M (2017). Cent Nerv Syst Agents Med Chem..

[5] Roa-Vidal N (2023). Int J Mol Sci..

[6] Mantovani A (2004). Trends Immunol..

[7] Martinez F.O (2008). Front Biosci..

[8] Ghafouri-Fard S (2021). Biomed Pharmacother..

[9] Wu X.Q (2016). Immunology..

[10] Wang L.X (2019). J Leukoc Biol..

[11] Ohnishi M (2023). J Neuroimmune Pharmacol..

[12] Hoover D.B (2017). Pharmacol Ther..

[13] Giunta S (2024). Geroscience..

[14] Chavda V.P (2024). Cells..

[15] Khakshooy A, Chiappelli F (2016). Bioinformation..

[16] Chiappelli F, Fotovat L (2022). Bioinformation..

[17] Giunta S (2024). Mech Ageing Dev..

[18] Khakshooy A, Chiappelli F (2024). Bioinformation..

[19] Tillman T.S (2023). ACS Chem Neurosci..

[20] Nadwa E.H (2023). Naunyn Schmiedebergs Arch Pharmacol..

[21] Kelly M.J (2022). Cell Rep Med..

[22] Fu J, Wu H (2023). Annu Rev Immunol..

[23] Chiappelli F (2025). Bioinformation..

[24] Schmitz CRR (2023). Front Immunol..

[25] Fotovat L (2024). Bioinformation..

[26] Penhaskashi J (2025). Bioinformation..

[27] Penhaskashi J (2023). Bioinformation..

[28] Zoheir N (2012). Inflamm Res..

[29] Bertolini M, Clark D (2024). Geroscience..

